# Elevated levels of urine isocitrate, hydroxymethylglutarate, and formiminoglutamate are associated with arterial stiffness in Korean adults

**DOI:** 10.1038/s41598-021-89639-w

**Published:** 2021-05-13

**Authors:** Ji-Hee Haam, Young-Sang Kim, Doo-Yeoun Cho, Hyejin Chun, Sang-Woon Choi, Yun Kyong Lee, Sang Wook Lim, Hyung Suk Koo, Moon Jong Kim

**Affiliations:** 1 Chaum Life Center, CHA University, 442, Dosan-daero, Gangnam-gu, Seoul, 06062 Republic of Korea; 2 Department of Family Medicine, CHA Bundang Medical Center, CHA University, 59 Yatap-ro, Bundang-gu, Seongnam-si, Gyeonggi-do 13496 Republic of Korea; 3 Division of Cardiology, Department of Internal Medicine, CHA Bundang Medical Center, CHA University, 59 Yatap-ro, Bundang-gu, Seongnam-si, Gyeonggi-do 13496 Republic of Korea; 4 Ahnkang Pain Free Hospital, 327, Eonju-ro, Gangnam-gu, Seoul, 06226 Republic of Korea

**Keywords:** Biomarkers, Cardiology

## Abstract

Recent evidence suggests that cellular perturbations play an important role in the pathogenesis of cardiovascular diseases. Therefore, we analyzed the association between the levels of urinary metabolites and arterial stiffness. Our cross-sectional study included 330 Korean men and women. The brachial-ankle pulse wave velocity was measured as a marker of arterial stiffness. Urinary metabolites were evaluated using a high-performance liquid chromatograph-mass spectrometer. The brachial-ankle pulse wave velocity was found to be positively correlated with l-lactate, citrate, isocitrate, succinate, malate, hydroxymethylglutarate, α-ketoisovalerate, α-keto-β-methylvalerate, methylmalonate, and formiminoglutamate among men. Whereas, among women, the brachial-ankle pulse wave velocity was positively correlated with cis-aconitate, isocitrate, hydroxymethylglutarate, and formiminoglutamate. In the multivariable regression models adjusted for conventional cardiovascular risk factors, three metabolite concentrations (urine isocitrate, hydroxymethylglutarate, and formiminoglutamate) were independently and positively associated with brachial-ankle pulse wave velocity. Increased urine isocitrate, hydroxymethylglutarate, and formiminoglutamate concentrations were associated with brachial-ankle pulse wave velocity and independent of conventional cardiovascular risk factors. Our findings suggest that metabolic disturbances in cells may be related to arterial stiffness.

## Introduction

Cardiovascular diseases (CVD) remain a major cause of mortality worldwide, which are mainly associated with atherosclerosis^1^. Recent studies revealed that arterial stiffness, as measured by pulse wave velocity (PAW), is associated with a powerful biomarker that indicates an increase in the likelihood of a future clinical event, disease recurrence, or progression of CVD^2–4^.

CVD has a complex etiology, with multiple risk factors and mechanisms contributing to its development. Hypertension, tobacco use, and high levels of cholesterol are significant risk factors for increased plaque burden and CVD^5,6^. In cells, various mechanisms and aberrations such as metabolic abnormalities, energy deficit, deregulation of autophagy, endoplasmic reticulum stress, and activation of apoptosis contribute to CVD pathogenesis^7^.

Detecting metabolites in the urine is one of the metabolic profiling technologies. The liquid chromatography-mass spectrometry (LC–MS) is used for the qualitative and quantitative determination of urine metabolites with very high sensitivity and specificity^8,9^. LC–MS is a combination of high-performance liquid chromatography (HPLC) and mass spectrometry (MS), which is widely used in pharmaceutical, chemical, and food applications ^8–11^. Many studies have reported that the levels of urine metabolites are significantly different in various diseases such as asthma^11^, autism spectrum disorder^12^, gestational diabetes mellitus^13^, jaundice^14^, prostate cancer^15^, purine and pyrimidine disorders^16^, and catecholamine-producing tumors^17^. One study revealed that plasma metabolites appear strongly correlated with PWV in women^18^. Among the urinary metabolites, the tricarboxylic acid cycle (TCA cycle) metabolites, also known as the citric acid cycle metabolites, were considered as byproducts of cellular metabolism important for the biosynthesis of nucleotides, lipids, and proteins^19–21^. Several studies revealed that urine metabolites are also associated with mitochondrial dysfunction^22–24^ and congenital mitochondrial diseases in children^25^.

Considering that cellular perturbations are related to CVD^7,26–28^, as a biomarker of cellular metabolism, urine metabolites may also be associated with CVD. However, the relationship of urine metabolomics analysis with clinical markers of CVD, such as brachial-ankle pulse wave velocity (baPWV), has not been comprehensively evaluated. Therefore, this study aimed to investigate the metabolomic signature according to baPWV to identify the novel metabolites associated with PWV and understand the molecular mechanisms underlying arterial stiffness.

## Results

### Characteristics of the study participants

The baseline characteristics of the participants are presented in Table 1. The mean age was 57.8 years. Among 330 subjects, 52.7% were men. The mean baPWV was 14.3 ± 3.0 m/s. Precisely, 63.9% of the subjects had significant physical activity and 19.7% had a metabolic syndrome.

**Table 1:** General characteristics of the subjects.

N = 330	
Age	57.8 ± 11.9
Sex (men)	174 (52.7%)
Current smoker	49 (14.8%)
Alcohol intake (g/week)	11.3 (0.0–64.7)
Physical activity	211 (63.9%)
Metabolic syndrome	65 (19.7%)
**Medical history with medications**	
Hypertension	81 (24.5%)
Diabetes	36 (10.9%)
Dyslipidemia	99 (30.0%)
**Anthropometry**	
Body mass index (kg/m^2^)	23.5 ± 3.1
Waist circumference (cm)	85.4 ± 8.9
Systolic BP (mmHg)	117.9 ± 14.5
Diastolic BP (mmHg)	74.7 ± 10.8
Pulse rate (bpm)	70.3 ± 9.7
Pulse wave velocity (m/s)	14.3 ± 3.0
**Laboratory results**	
Fasting glucose (mmol/L)	4.88 (4.44–5.33)
Total cholesterol (mmol/L)	5.03 ± 0.94
Triglyceride (mmol/L)	1.02 (0.73–1.46)
HDL cholesterol (mmol/L)	1.43 ± 0.33
GFR (mL/min/1.73 m^2^)	79.5 ± 16.8
**Organic acid profile (μg/mg Cr)**	
Adipate	0.86 (0.62–1.28)
Suberate	0.85 (0.56–1.26)
Ethylmalonate	2.51 (1.92–3.20)
Pyruvate	1.07 (0.55–1.91)
l-Lactate	12.9 (9.2–18.0)
β-Hydroxybutyrate	2.12 (1.07–4.84)
Citrate	636.8 (468.6–851.6)
cis-Aconitate	46.8 (36.7–59.3)
Isocitrate	75.0 (58.5–94.2)
α-Ketoglutarate	14.8 (7.8–25.3)
Succinate	4.07 (2.39–6.38)
Fumarate	0.25 (0.15–0.47)
Malate	0.48 (0.29–0.90)
Hydroxymethylglutarate	3.52 (2.58–4.61)
α-Ketoisovalerate	0.16 (0.10–0.25)
α-Ketoisocaproate	0.19 (0.12–0.27)
α-Keto-β-methylvalerate	0.75 (0.49–1.17)
Xanthurenate	0.49 (0.38–0.66)
β-Hydroxyisovalerate	5.17 (3.79–6.75)
Methylmalonate	1.33 (0.98–1.78)
Formiminoglutamate	0.63 (0.45–0.90)

Data are expressed as mean ± SD, median (interquartile range), or number (proportion); GFR determined using the Modification of Diet in Renal Disease method.

*BP* blood pressure, *HDL* high-density lipoprotein, *GFR* glomerular filtration rate.

### Association of urine metabolites and baPWV

The correlation of baPWV with urine metabolites was evaluated (Table 2). Among men, the levels of l-lactate, citrate, isocitrate, succinate, malate, hydroxymethylglutarate, α-ketoisovalerate, α-keto-β-methylvalerate, methylmalonate, and formiminoglutamate were positively associated with baPWV. Among women, the levels of cis-aconitate, isocitrate, hydroxymethylglutarate, and formiminoglutamate were positively associated with baPWV.

**Table 2:** The Spearman correlation coefficients of the levels of the metabolites with baPWV.

	Total		Men		Women	
	rho	p	rho	p	rho	p
Adipate	− 0.046	0.400	− 0.062	0.409	0.016	0.839
Suberate	0.031	0.566	0.034	0.651	0.040	0.612
Ethylmalonate	0.080	0.143	0.133	0.078	0.099	0.216
Pyruvate	− 0.079	0.146	− 0.038	0.612	− 0.114	0.154
l-Lactate	− 0.027	0.626	0.176	0.019*	− 0.135	0.091
β-Hydroxybutyrate	0.038	0.489	0.136	0.071	0.007	0.935
Citrate	0.142	0.009*	0.219	0.003*	0.151	0.058
cis-Aconitate	0.086	0.117	0.111	0.140	0.164	0.039*
Isocitrate	0.157	0.004*	0.162	0.031*	0.238	0.002*
α-Ketoglutarate	− 0.043	0.430	0.043	0.570	0.030	0.711
Succinate	0.072	0.187	0.164	0.030*	0.087	0.274
Fumarate	0.084	0.125	0.162	0.031*	0.083	0.300
Malate	0.118	0.030*	0.254	< 0.001*	0.084	0.292
Hydroxymethylglutarate	0.212	< 0.001*	0.217	0.004*	0.323	< 0.001*
α-Ketoisovalerate	0.135	0.013*	0.156	0.038*	0.117	0.142
α-Ketoisocaproate	0.031	0.574	0.136	0.071	− 0.057	0.473
α-Keto-β-methylvalerate	0.114	0.037*	0.157	0.037*	0.086	0.280
Xanthurenate	0.109	0.046*	0.061	0.422	0.144	0.069
Β-Hydroxyisovalerate	− 0.002	0.977	0.023	0.766	− 0.011	0.894
Methylmalonate	0.107	0.049*	0.163	0.030*	0.146	0.066
Formiminoglutamate	0.384	< 0.001*	0.356	< 0.001*	0.415	< 0.001*

*BaPWW* Brachial-ankle pulse wave velocity.

Rho indicates Spearman correlation coefficients.

*P < 0.05.

### The distribution of the levels of key metabolites with baPWV

**Figure 1:**
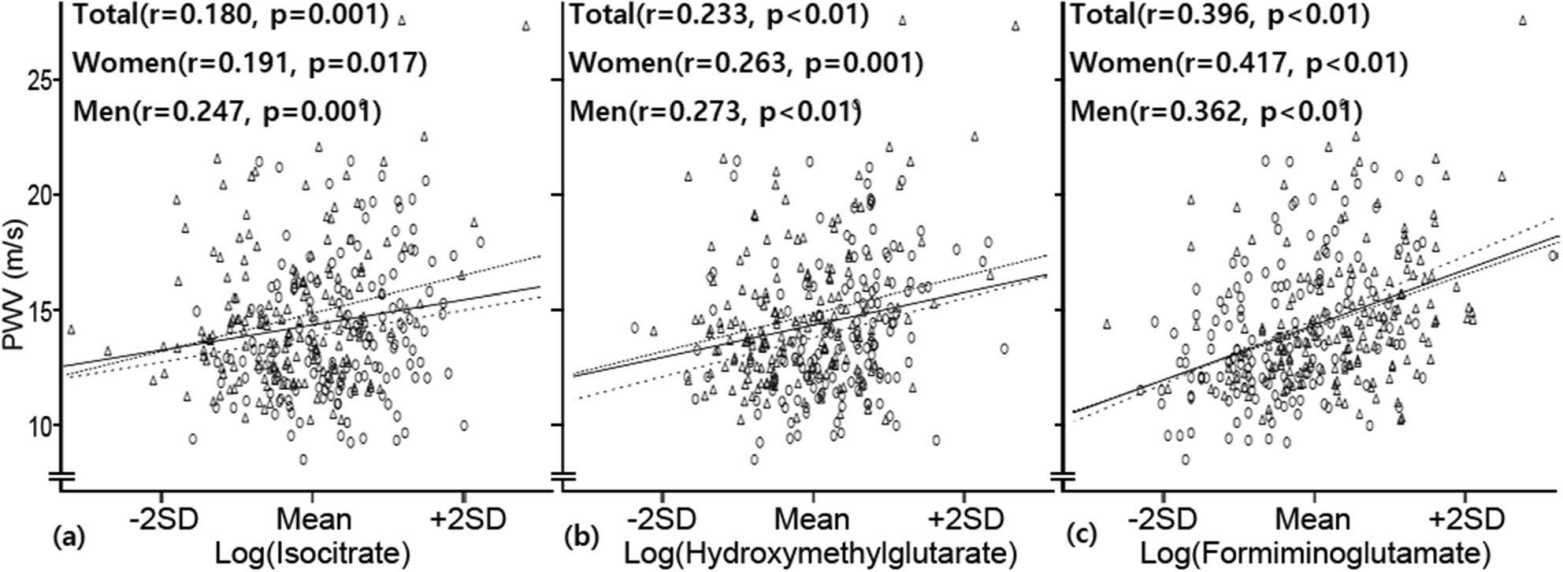
The Scatter plots of the levels of key metabolites with baPWV. Each concentration of the metabolites (isocitrate (**a**), hydroxymethylglutarate (**b**) and formiminoglutamate (**c**)) is logarithmically transformed and standardized. Men are shown as triangles, and women are displayed as circles. Solid lines are fit for all subjects; dense dotted lines are fit for men, and sparse dotted lines are fit for women. r indicates Pearson correlation coefficients. *BaPWV* brachial-ankle pulse wave velocity, *PWV*, pulse wave velocity.

We selected isocitrate, hydroxymethylglutarate, and formiminoglutamate, which were significantly associated with baPWV. The distribution of the levels of three urine metabolites (isocitrate, hydroxymethylglutarate, and formiminoglutamate) according to baPWV is shown in Fig. 1. Figure 1 shows the positive correlation of isocitrate, hydroxymethylglutarate and formiminoglutamate with baPWV.

### Standardized regression coefficients of the levels of key metabolites for baPWV

**Figure 2:**
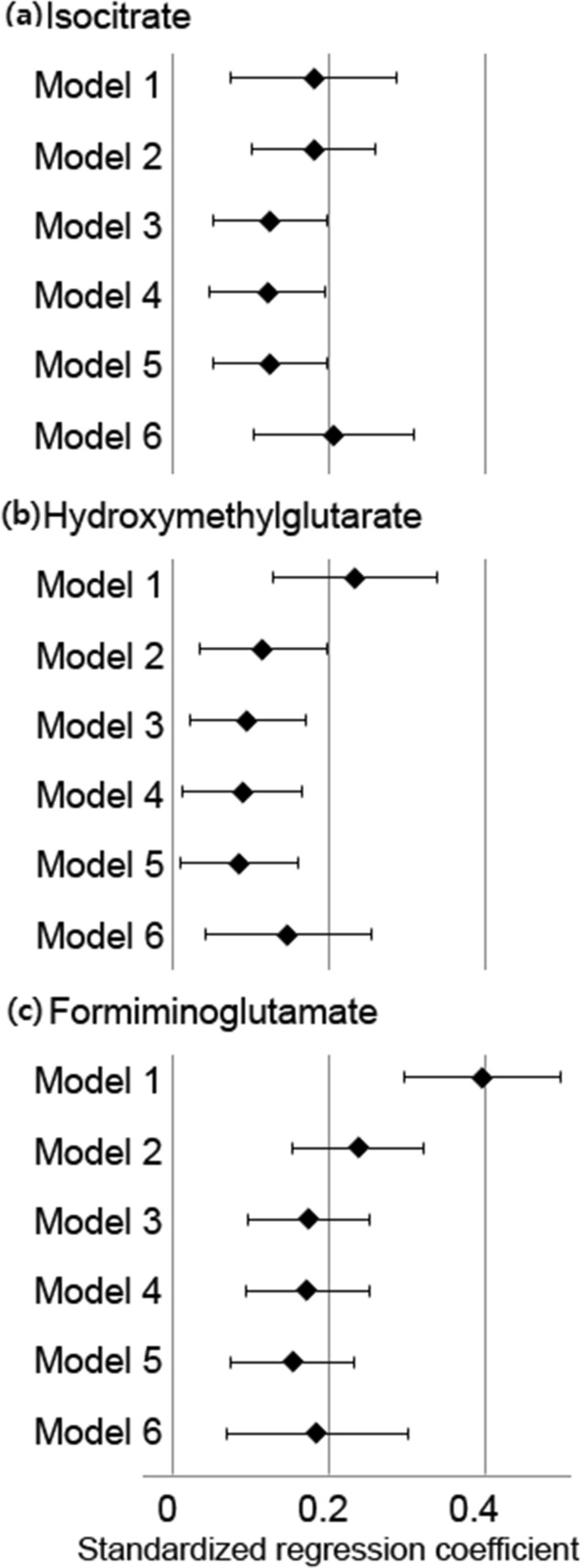
Standardized regression coefficients of the levels of key metabolites for baPWV. The concentrations of the key metabolites (isocitrate (**a**), hydroxymethylglutarate (**b**) and formiminoglutamate (**c**)) are logarithmically transformed and inserted in the regression models. Model 1 constitutes crude models. Model 2 additionally includes the variables of age and sex. Model 3 additionally includes variables of mean BP and heart rate. Model 4 additionally includes variables of total cholesterol, HDL cholesterol, BMI. Model 5 additionally includes the following variables: medication history of hypertension, diabetes, and dyslipidemia, habits of smoking, significant alcohol consumption, and physical activity. In Model 6, the subjects with history of medications, smoking, and significant alcohol consumption were excluded. The covariates in Model 6 were same as those included in Model 4. Error bars show standard error of the means. *BaPWV* brachial-ankle pulse wave velocity, *BP* blood pressure, *HDL* high-density lipoprotein, *BMI* body mass index.

The relationship between baPWV and the levels of urine metabolites was assessed after adjusting for potential confounders (Fig. 2). In the crude model (model 1), positive relationships of baPWV with the levels of isocitrate, hydroxymethylglutarate, and formiminoglutamate were observed (*P* = 0.001, *P* < 0.001, and *P* < 0.001, respectively). After the adjustment for all the confounders (age, sex, mean blood pressure (BP), heart rate, total cholesterol, high-density lipoprotein (HDL) cholesterol, alcohol history, physical activity, smoking history, and medication history of hypertension, diabetes, and dyslipidemia), the positive relationship between baPWV and the three metabolites (isocitrate, hydroxymethylglutarate, and formiminoglutamate) levels was significant (*P* = 0.001, *P* = 0.029, and *P* < 0.001, respectively) (model 5). In the subjects excluding those with hypertension, diabetes, or statin use, and alcohol and smoking history (N = 151), the levels of isocitrate, hydroxymethylglutarate, and formiminoglutamate were significantly related to baPWV (*P* < 0.001, *P* = 0.005, and *P* = 0.003, respectively) (model 6).

## Discussion

In this cross-sectional study of 330 men and women, higher levels of urine isocitrate, hydroxymethylglutarate, and formiminoglutamate were significantly associated with high baPWV after adjusting for conventional cardiovascular (CV) risk factors.

Previous studies have demonstrated that cellular mechanisms, including intracellular hyperglycemia, increasing fatty acid flux and oxidation, and mitochondrial dysfunction, play a role in coronary atherogenesis and cardiomyopathy^7,29–31^.

One of the cellular mechanisms, higher fatty acid flux and oxidation, is related to various heart diseases^30,31^. During cardiac ischemia, elevated rates of fatty acid oxidation result in the inhibition of glucose oxidation by inhibiting the activity of pyruvate dehydrogenase leading to increased production of l-lactate^31^. As in previous reports, higher lactate levels were related to cardiac problems such as heart failure, endocardial damage, and cardiac ischemia ^32,33^. In this study, l-lactate levels were positively correlated with baPWV in men, before adjusting for confounders. However, we did not find any significant correlation of l-lactate levels with baPWV after adjusting for confounders. This discrepancy may have arisen from our small sample size and non-homogeneous study population. Further research is needed to investigate the relationship of l-lactate and arterial stiffness in a large population.

Among the cellular perturbations, mitochondrial dysfunction also plays a central role in the development of CVD^7,26,29,30,34^. Mitochondria contribute in oxygen sensing, cellular signaling, cell stress regulation, and energy production. The mitochondrial respiratory chain is the major pathway of energy production. Mitochondria are very sensitive to nutrient and oxygen supply and can adapt to a changing environment. When this adaptation is impaired, it leads to a progressive decline of mitochondrial function, which is associated with abnormalities in the respiratory chain and ATP synthesis, increased oxidative stress, and activation of signaling proteins^35^. Reactive oxygen species (ROS) overproduction then leads to the oxidation of lipids and proteins and promotes atherogenesis by inducing endothelial dysfunction, vessel inflammation, and accumulation of oxidized low-density lipoprotein (LDL)^28^. The diagnosis of mitochondrial dysfunction relies on a combination of clinical presentation, measurement of metabolites, and analysis of respiratory chain function^22,36^. Alban et al. showed the relationship between mitochondrial respiratory chain activities and urine metabolites in 75 cases^24^. Another previous study indicated that mitochondrial dysfunction is accompanied by the excretion of citric acid cycle intermediates^22^. Elevated levels of one of the citric acid cycle intermediates in urine, isocitrate, may reflect mitochondrial nicotinamide adenine dinucleotide phosphate (NADP) + -isocitrate dehydrogenase inactivity. Mitochondrial NADP+-isocitrate dehydrogenase catalyzes the oxidative decarboxylation of isocitrate, producing alpha-ketoglutarate, and regulates cardiomyocyte energy and redox status. Some studies suggest that mitochondrial NADP+-isocitrate dehydrogenase dysfunction contributes to the etiology of cardiomyopathy^37,38^, cancer^39^, and cell damage after kidney ischemia–reperfusion injury^40^. In our study, we demonstrated that urine isocitrate levels were significantly associated with baPWV and was still significantly correlated with baPWV after adjusting for confounders.

Regarding citric acid cycle intermediates, high levels of hydroxymethylglutarate may reflect the inadequate endogenous synthesis of CoQ10^41,42^. Some studies have demonstrated that CoQ10 might play a role in the prevention of heart ailments, inhibition of LDL oxidation, and progression of atherosclerosis^43^. Consistent with previous studies, our results reveal the relationship between baPWV and urine hydroxymethylglutarate; after the adjustment of confounders, urine hydroxylmethylglutarate levels were still significantly associated with baPWV.

Succinate is a metabolic intermediate of the TCA cycle within cells, like hydroxymethylglutarate^44^. In mitochondria, succinate is produced as an intermediate metabolite formed from the conversion of succinyl-CoA and is oxidized by succinate dehydrogenase (SDH) to form fumarate. Meanwhile, succinate is also produced from succinic semialdehyde via the γ-aminobutyric acid (GABA) shunt^44^. Generally, succinate is considered an intracellular metabolite, but succinate also has been shown to accumulate in the extracellular tissue environments related to the conditions of stress and inflammation. For instance, in microbial fermentation, succinate is also formed by the reversal of partial TCA cycle reactions^45–47^. In this study, urine hydroxymethylglutarate levels were significantly associated with baPWV, but the same was not true for urine succinate. Although hydroxymethylglutarate and succinate are in close connection in the TCA cycle, the mechanisms for succinate release are diverse and unclear, which resulted in the different results.

Formiminoglutamic acid is an intermediate metabolite in the degradative conversion of histidine to glutamic acid. An increase in urinary formiminoglutamic acid levels may be seen in patients with folic-acid deficiency, vitamin B12 deficiency, and liver disease^48,49^. Some studies showed that folate deficiency is associated with heart disease^50^ and CVD^51,52^. Folate supplementation delays the development of atherosclerotic lesion by modulating monocyte chemotactic protein-1 (MCP-1) and vascular endothelial growth factor (VEGF) DNA methylation levels^53^. In our study, urinary formiminoglutamate levels were positively correlated with higher baPWV, independent of confounders.

Our study has several limitations. First, our study was cross-sectional; thus, we could not demonstrate a causative relationship between urine metabolites and baPWV. Second, as noted earlier, we had a small sample, and our population was considerably heterogeneous; the subjects studied had various underlying diseases and a wide difference in age. Third, we lacked sufficient disease history of the subjects which might be related to arterial stiffness. Finally, we did not consider the day by day variability in urine measurement and did not reflect the long-term data. Additional long-term studies about the metabolic status can help overcome this limitation.

In conclusion, our study reveals that higher concentrations of urine isocitrate, hydroxymethylglutarate, and formiminoglutamate were positively correlated with higher baPWV, independent of conventional CV risk factors. Our findings show that the changes in these metabolites were associated with arterial stiffness. Further studies are required to reveal the causal relationship between urine metabolites and baPWV. This may serve as a new useful clinical biomarker for predicting elevated arterial stiffness and may help understand the molecular mechanisms underlying arterial stiffness.

## Methods

### Study population

The participants enrolled in this cross-sectional study included adults who visited the Chaum Life Center, CHA University, Seoul, Republic of Korea. Among all adults who attended the periodic medical check-up from November 2016 to December 2018, 927 participants agreed to participate in the study and had undergone urine metabolomic analyses. Subjects were required not to take supplements, including vitamin and herbs, for at least 1 week. We excluded 591 subjects who had not undergone measurements of baPWV. Subjects with renal disease, malignant disease, thyroid disease, collagen diseases, or infections, and acute disease, abnormal liver function, and a history of stroke, angina, or myocardial infarction were also excluded. Finally, 174 men and 156 women were enrolled in our study. This study was approved by the institutional review board of CHA Bundang Medical Center. The participants provided informed consent prior to enrollment. All the procedures were carried out in accordance with the relevant guidelines.

### Medical history, metabolic syndrome and lifestyle habits

The medical history, medication, and lifestyle habits of the subjects were collected. The presence of metabolic syndrome (MetS) was defined by the National Cholesterol Education Program Adult Treatment Panel III criteria^54^. The cutoff values for central obesity were applied in accordance with a well-validated previous Korean study^55^. The MetS was defined by the presence of three or more of the following components: (1) waist circumference ≥ 90 cm for man and ≥ 85 cm for women, (2) systolic BP ≥ 130 mmHg or diastolic BP ≥ 85 mmHg or antihypertensive medication use, (3) HDL cholestrol < 1.04 mmol/L for men and 1.29 mmol/L for women, (4) triglyceride > 1.69 mmol/L, and (5) elevated fasting blood glucose ≥ 6.11 mmol/L or taking hypoglycemic agents.

Patients were categorized as non-smokers or current smokers based on their smoking habits. Significant alcohol consumption was defined as > 21 standard drinks/week in men and > 14 standard drinks/week in women over a 2-year period^56^. Significant physical activity was defined as ≥ 3 exercise/week.

### Anthropometric measurements

Height and weight were measured in centimeters and kilograms, respectively, using standardized protocols while the subjects were dressed in light clothing and had their shoes off. Body mass index (BMI) was calculated from height and weight. BP was measured after resting for 10 min in a sitting position using an automatic sphygmomanometer (TM-2655P, A&D Company, Tokyo, Japan) with an appropriate cuff size. The mean BP calculated using a formula in which the diastolic BP is doubled and added to the systolic BP and the composite sum was divided by 3^57^.

### Biochemical measurements

Blood samples were collected and subsequently analyzed at a central certified laboratory at the CHA Gangnam Medical Center. Blood samples were collected from the antecubital vein early in the morning after an 8-h overnight fast. Fasting plasma glucose, high-density lipoprotein (HDL) cholesterol, and triglyceride levels were measured using a chemistry autoanalyzer (Hitachi 7600-110, Tokyo, Japan). Glomerular filtration rate (GFR) determined using the Modification of Diet in Renal Disease method^58^.

### Measurements of baPWV

BaPWV was measured using a non-invasive vascular screening device (VP-1000 plus, Omron Healthcare, CA, USA). Subjects were examined in the supine position for at least 5 min, with elbows and ankles fastened to the blood pressure cuff. Electrocardiographic electrodes sheet and telepathy were attached on both wrists and placed at the second intercostal space at the left margin of the sternum. These values were measured after individuals had rested for at least 5 min. We adopted the average value of the left and right baPWV.

### Measurements of urine metabolites

After 8-h fasting, the urine samples were collected from each subject and promptly placed in a freezer. Urine samples were analyzed at the Eone Laboratory, Inc. (Yeonsu-gu, Incheon, Republic of Korea). Fasting samples are usually used to explore how systemic metabolism differs between populations with different dietary habits^59,60^. The creatinine concentrations of the urine samples were determined before the analysis, and urinary concentrations of metabolites were normalized with urine creatinine to minimize the variability in urine concentrations.

The urine samples (300 μL) of the subjects were transferred into the autosampler vial. The samples were prepared according to the standardized protocol and injected into the HPLC–tandem mass spectrometry (HPLC–MS/MS) system. The HPLC–MS/MS analyses were conducted with AB Sciex Triple Quad 4500 MD (Framingham, MA, USA) with an electrospray ionization interface. The quantification was performed using an Organic Acids Urine LC–MS/MS analysis kit (ZIVAK, Kocaeli, Turkey). Injection volumes were 20.0 μL, and the flow rate throughout the analysis was 0.25 mL/min. The selected reaction monitoring transitions and the related optimized declustering potential, collision energy, and collision cell exit potential for the different analytes were determined according to the manufacturer’s manual. For all metabolites, a standard curve of known concentrations consisting of three concentrations was built. Quantification of metabolites in samples was based on these standard curves and carried out automatically with the MS controller MultiQuant MD 3.0.2.

### Statistical analysis

SPSS version 25.0 (IBM, Armonk, NY, USA) was used to perform all data analyses. All continuous variables are reported as means ± standard deviations (SDs) or median (interquartile range). The categorical variables are expressed as number (percentage). We converse normalized the metabolite data, as the metabolite concentrations were not normally distributed. To identify the metabolites that associate with PWV, Spearman correlation analysis of the metabolite levels with baPWV was performed.

The metabolites that were significantly related to baPWV in both men and women were considered as key metabolites. We drew scatter plots to show the association between key metabolites and baPWV. Fit lines were also drawn in the plots.

To avoid the effects of the confounding factors, associations of metabolites with urine metabolites were tested using covariate-adjusted multivariable regression to examine the relationship between urine metabolites and baPWV with the adjustment of confounding factors. Model 1 means a crude model. First, demographic factors, age, and sex were included in model 2. Then, mean BP and heart rate were additionally included in Model 3 as strongly influencing factors for PWV. Total cholesterol, HDL cholesterol levels, and BMI, which are conventional CV risk factors, were additionally included in model 4. As factors that may potentially affect PWV, medication history of hypertension, diabetes mellitus, and dyslipidemia, smoking and alcohol history, and physical activity were additionally included in model 5. To minimize the effects of the medications for hypertension, diabetes mellitus, and dyslipidemia and smoking and alcohol history, the regression models were analyzed again in the subjects without a medication history of hypertension, diabetes mellitus, or statin use, and alcohol and smoking history (model 6). For all analyses, a P-value < 0.05 was considered statistically significant.
